# Post-vaccination evaluation of an erysipelas/parvovirus bivalent vaccine administered to sows during lactation on follicular development and piglet growth

**DOI:** 10.1016/j.vas.2025.100442

**Published:** 2025-03-15

**Authors:** E. Sánchez-Tarifa, FA. García-Vázquez, A. Vela, L. Sanjoaquín, MV. Falceto, A. Fernández-Fontelo, O. Gómez-Duran, C. Alonso, I. Hernández-Caravaca

**Affiliations:** a Department of Physiology, Faculty of Veterinary Science, University of Murcia, Murcia, Spain; b Thinkinpig SLP. Zaragoza, Spain; c Department of Animal Pathology, Agroalimentary Institute of Aragon IA2, University of Zaragoza-CITA, Spain; d Departament de Matemàtiques, Universitat Autònoma de Barcelona, Barcelona, Spain; e School of Business and Economics, Humboldt-Universität zu Berlin, Berlin, Germany; f Boehringer Ingelheim Vetmedica GmbH, AH Swine, Ingelheim, Germany; g Department of Community Nursing, Preventive Medicine and Public Health and History of Science, University of Alicante, Sant Vicent del Raspeig, Alicante, Spain

**Keywords:** *Erysipelothrix rhusiopathiae*/parvovirus, Vaccination, Swine folliculogenesis, Offspring performance, Sow feed consumption

## Abstract

Vaccines against *Erysipelothrix rhusiopathiae* (ER) and porcine parvovirus (PPV) are employed in sow farms worldwide. This study evaluated the impact of administering a bivalent ER/PPV vaccine during lactation. Multiparous sows (*n* = 83) of two different farms (A (herd size 900 sows) and B (1000 sows)) were allocated in two different groups: 1) sows inoculated with saline solution (non-vaccinated group); 2) sows inoculated with the bivalent vaccine against ER/PPV (vaccinated group). The injections were administered at ∼11 days of lactation. The study was conducted in four different experiments: the first experiment, evaluating the female genital tract (uterus and ovary) and follicle development, was performed in culled sows (*n* = 19) of Farm A the day after weaning (∼24 days). The remaining experiments, which examined internal temperature, offspring growth, and sow feed intake, were conducted at Farm B (*n* = 64). The results of follicle populations, showed a greater frequency of large follicles in non-vaccinated compared to vaccinated sows was observed (*p* < 0.001). The final weight of the piglets and their weight gain were higher in the non-vaccinated group (*p* ≤ 0.05). A statistical trend was also observed in the average weight gain of piglets. No differences were observed regarding sow´s rectal temperature between groups around the period of injection but the vaccinated group consumed less sow feed on the day of injection (*p* = 0.016). In conclusion, the administration of this vaccine against ER/PPV during the lactation period had a negative impact on follicle development, sow feed intake, and offspring weight gain and final weight.

## 1 Introduction

Erysipelas in animals is caused by infection with the Gram-positive bacteria *Erysipelothrix spp. Erysipelothrix spp*. is divided into eight different species which includes *Erysipelothrix rhusiopathiae* (ER) (Skerman et al., 1980; Buchanan, 1918) belonging to the genus Erysipelothrix in the family Erysipelothrichaceae. Pigs are considered the most important reservoir for ER. It was estimated that 30–50 % of healthy pigs carry the bacteria in tonsils and lymphoid tissues (Steppson & Berman, 1978). Clinically, Erysipelas is characterized by sudden death, pyrexia, red skin patches (red sick-ness), arthritis or cardiac insufficiency (reviewed by Opriessnig et al., 2020). Moreover, reproductive performance is also compromised when Erysipelas is present, being associated with vulval discharges, low fertility, abortions or smaller litter size (Bilkei, 1996; Hoffmann & Bilkei, 2002). So, this bacterium has an important economic influence at different levels of swine industry and can impact all pig production levels such as breeding herd, grow-finish farm or slaughterhouse (reviewed by Opriessnig et al., 2020), reasons why the majority of pig breeding are vaccinated. Essentially, the commercial vaccines available are based in attenuated erysipelas and bacterins. In gilts a common vaccination schedule consists in the i.m. vaccination of the gilts at 22 and 26 weeks of age and ahead of the first insemination. Subsequent revaccinations in multiparous sows are applied in each reproductive cycle 10 days after farrowing because the protective immunity is around 4–6 months (reviewed by Opriessnig et al., 2020).

On the other hand, porcine Parvovirus (PPV) is a virus (family Parvoviridae, Parvovirinae subfamily, and Ungulate protoparvovirus 1 species) present in the majority of pig herds worldwide (van den Born et al., 2020) and considered to be one of the major causes of reproductive failure (Streck & Truyen, 2020). PPV does not cause clinical signs in adults, so this disease can be considered when females return to oestrus with no apparent reasons or delay in parturitions with an increase in the number of mummified fetuses and small litters (Streck & Truyen, 2020), because fetuses infected early in pregnancy usually die, resulting in their mummification or resorption (van den Born et al., 2020). PPV is controlled by a proper management of internal and external biosecurity of the herd and through vaccination. Current commercial PPV vaccines consist of inactivated forms of the original virus (Truyen & Streck, 2012) or recombinant virus (García-Morante et al., 2019). In the case of reproductive females, the vaccination protocols are similar to the ones explained for ER (Truyen & Streck, 2012), because in most of the times the antigens are mixed by the pharmaceutical industry in the same vaccine. As mentioned, vaccines against erysipelas and PPV are routinely used in the pig industry. For user convenience and to reduce the number of injections given to sows in the breeding herd during the lactation period, a typical immunization schedule usually includes the combination of two vaccines as a ready to use product. This approach improves both animal welfare and the labor efficiency of farmers (Sánchez-Matamoros et al., 2019). However, it is also useful for pig health to improve the comfort of the breeding environment because pigs exposed to stress can reduce their immunological responses against pathogens (Morimoto et al., 2022). Certainly, any situation of stress during the lactation period may have an impact on their productivity (feed consumption, milk production, growth of the current litter), risking their maximum productive level or even having a negative impact in the following reproductive cycle. Numerous factors influence the proper development of follicles in sows. These include season, parity, body condition, stress and lactation duration. These factors are considered primary influences on ovarian follicular growth in sows (Quesnel et al., 2005; Peltoniemi et al., 2006; Auvigne et al., 2010; Bertoldo et al., 2011; Knox et al., 2019). In the sows, waves of follicular growth are absent (Lucy et al., 2001). During the luteal and early follicular phases, approximately 50 follicles measuring 1–6 mm are present (Foxcroft et al., 1985). Subsequent to the elimination of inhibitory effects associated with suckling at weaning, follicles that exceed 4 mm (dominant follicles) undergo further growth, while the number of small follicles diminishes and begin atresia (Hafez, 2000). It is important to note, that sows showing smaller follicles at weaning had showed longer intervals for estrus and ovulation and smaller litters at farrowing (Kemp et al., 2005; Lopes et al., 2014; TP Lopes et al., 2020).

Nowadays, vaccines have evolved into safer products noticing their use not only during the lactation period but in a wider range of periods of the reproductive cycle of the sow. Nevertheless, there are no detailed published data about the impact of ER/PPV vaccination on reproductive tract and productive performance with the routinely used schedules. According to the pointed out information, the objective of the present study was to evaluate the effect of the ER/PPV vaccination on sows during lactation, with particular regard to its impact on the reproductive tract, temperature, and feed consumption of the sow, as well as on their offspring (piglets' growth).

## 2 Materials and methods

### 2.1 Study design

**Fig. 1:**
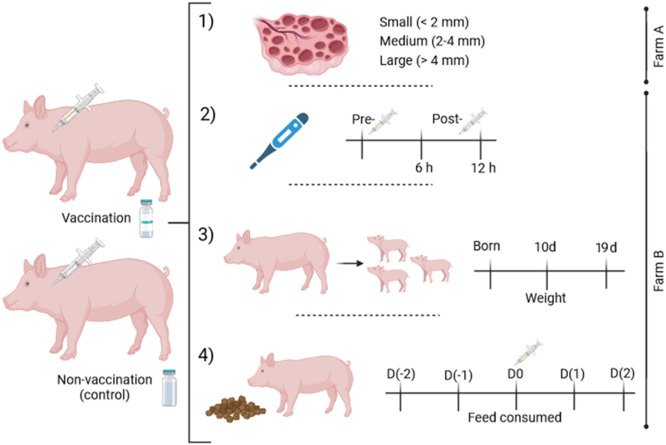
Scheme of the experimental design. Two groups of sows were allocated in one of the two experimental groups: vaccinated vs. non-vaccinated (control group injected with saline solution). The vaccination was performed ∼11 days of lactation. Experiment 1) Reproductive tracts of weaned sows were recovered from the slaughterhouse and follicles were counted and classified depending on their size (small, medium, or large). Experiment 2) The temperature of the sows was evaluated before vaccination (pre-vaccination) and after vaccination (6 h and 12 h post-vaccination). Experiment 3) The weight of the piglets was analyzed at born, 10 days of age, and before weaning (19 days of age). Moreover, ADWG and total weight gain were calculated. Experiment 4) The feed consumed by the sows of the study was evaluated before (Day(−2) and D(−1)) vaccination, the day of vaccination (D0), and after vaccination (Day(1) and D(2)).

The design of the study (Fig. 1) was directed to evaluate the impact of the bivalent vaccine (ER/PPV) administered in post-farrowing sows. Concretely, sows were vaccinated (vaccinated group) or injected with sterile physiological saline solution (2 ml) (non-vaccinated) during lactation (∼11 days of lactation in all the experiments) in late summer early autumn. The sows included in the first experiment (experiment 1), were a total of 19 sows (Farm A) of the same batch distributed in non-vaccinated (*n* = 9, parity= 6.88 ± 0.78) and vaccinated sows (*n* = 10, parity= 6.60 ± 1.07). They were sacrificed the day after weaning (24.4 ± 4.37 days after farrowing). The follicles of the ovaries were classified according to their size in (i) small (< 2 mm in ø), (ii) medium (2–4 mm in ø), and (iii) large (> 4 mm in ø). Moreover, the weight of the ovaries (g), uterine horns (g), oviducts (g), and diameter of uterine arteries (cm) were recorded.

In experiment 2, 64 sows (Farm B) of the same batch were distributed in vaccinated (*n* = 37, parity = 3.85 ± 2.98) and non-vaccinated (*n* = 27, parity = 3.88 ± 2.80) groups having similar reproductive performance (see Table 3). In both groups, we took the rectal temperature of the sows (pre-vaccination and 6 h and 12 h post-vaccination), and weighted the piglets (at birth, at 10 e and at 19 days of age) and the calculated the (ADWG of piglets from birth to final weight at 19 days of lactation (experiment 3). Finally, the feed consumed by the sows [before (Day(−2) and D(−1)) vaccination, the day of vaccination (D0) and after vaccination (Day(1) and D(2))] were evaluated (experiment 4).

### 2.2 Animals and housing

A total of 83 crossbred sows [Large-White X Landrace] from two commercial sow farms were used for this study. Farm A consisted of a 900-sow farrow-to-nursery (Genetic Topigs Norsvin, TN70), whereas Farm B comprised 1000-sows in a farrow-to-wean intensive conventional system (Genetic Geneplus Iberia, S.L). Both farms were in Spain's North-eastern area, with an industry-standard biosecurity program in place certified by the herd veterinarian. Sows on the farms were actively mass vaccinated against the porcine respiratory and reproductive syndrome virus (PRRSv) every four months, E. coli and Clostridium perfringens type C (bivalent vaccine) in last trimester of every gestation and against Parvovirus and *E. rhusiopathiae* (bivalent vaccine) during the lactation period in each cycle. Both farms were stable against PRRSv based on the classification system developed by the American Association of Swine Veterinarians (Holtkamp et al., 2011). It is noteworthy that throughout the course of the study's development, no occurrence of disease outbreaks or alterations in animal diets occurred.

During the trial, animals were allocated in farrowing crates (according to the European Commission Directive for Pig Welfare).

### 2.3 Vaccine

Sows used in this trial received vaccination against ER and PPV bivalent vaccine following manufacture´s protocol [Eryseng® Parvo, HIPRA, Amer, Spain]. The vaccine's adjuvant is alum, the most widely used vaccine adjuvant due to its safety, cost-effectiveness, and ability to enhance the body's immune response to various antigens. (Di Benedetto et al., 2024). The treatment was administered intramuscular (i.m.) into the neck musculature caudal of the ear base and at a volume of administration of 2 ml per animal. All injections were performed by a dedicated worker not involved with data collection.

### 2.4 Uterine tract postmortem examination

The experiment was conducted using only those sows that had been selected for culling as part of the farm's standard culling procedure following weaning. None of the selected sows exhibited any reproductive problems in their reports prior to culling. The genital tract of the sows (ovarian structures, oviduct, uterine horns, uterine arteries) was evaluated the day after weaning. Measurements of ovarian follicle size (right and left) and uterine artery size were performed with a digital caliber (RS PRO) by an expert under single-blind study conditions. The weight (scale in grams-g) of the ovaries, oviducts, and uterine horns was recorded with a precision digital scale (Nahita 5041).

### 2.5 Insemination procedure, pregnancy diagnosis, farrowing and litter performance

The insemination was carried on after estrus detection, that was performed twice daily by experienced workers by allowing sows nose-to-nose contact with mature boars and applying back pressure. The occurrence of estrus was defined by the standing reflex in front of a teaser boar and reddening and swelling of the vulva. Only sows with clear signs of estrus were used for the experiment. The post-cervical artificial insemination procedure was performed as described by Hernández-Caravaca et al. (2012). Pregnancy was confirmed by ultrasound 23–28 days after insemination by transabdominal ultrasonography (Echoscan T-300 S, Barcelona, Spain). At 110 days of gestation, pregnant sows were moved from the gestation facilities to the farrowing pens. Farrowing data were recorded for each sow. At the end of farrowing, farrowing rate (%) and the number of piglets born were recorded. Each piglet was classified as piglet born alive, dead, or mummified. The weaning age of the piglets in both farms was ∼24 days. In experiment 2, alive piglets were identified with eartags and were assigned to their mother sow at birth for precise recording. During the lactation period, crossfostering was allowed within each group. Individual body weights (kg) of the piglets were collected on the day of birth and a few days before weaning using a digital handing scale. Moreover, piglet average daily weight gain (ADWG) was obtained for the surviving piglets (calculated from the day of birth to the day of the last weighing at 19 days after birth). The piglets were provided with an ad libitum supply of commercial diets commencing on the sixth day of lactation.

### 2.6 Rectal temperature

Temperatures were measured rectally in the sows using self-calibrated digital thermometers at approximately the same time each day and recorded in degrees Celsius (°C) units. Pyrexia was defined as an increase in temperature of >1.5 °C respective to the baseline temperature of the individual animal (pre-vaccination temperature) (Noguera et al., 2021).

### 2.7 Feed intake

Daily feed intake data were individually obtained by sow using control of feeding consumption devices (GESTAL SOLO, JYGA Technologies, Quebec, Canada). Feed was available ad libitum with commercial diets for lactating sows.

### 2.8 Statistical analysis

Statistical analyses were performed with the SPSS 24.0 software package (IBM SPSS Inc., Chicago, IL, USA). Sow characteristics parameters were analyzed for normality by a Shapiro–Wilk test, in which showed that all parameters had a normal distribution. The Chi-squared test was used to evaluate the distribution of follicles within different follicle size classes between both experimental group (vaccinated vs non-vaccinated). The proportion of each follicle category (small, medium, large) between non-vaccinated and vaccinated animals was compared by *t*-test (data with normal distribution; small and large categories) or Wilcoxon test (data with non-normal distribution; medium category). The results are represented as the mean ± SD (standard deviation) or mean ± SEM (standard error of the mean) accordingly. Values were considered significantly different when *p* ≤ 0.05. A statistical trend was considered when *p* > 0.05 and *p* < 0.07. The correlation between the number of follicles (small, medium, and large) and ovary weight and uterine artery size was assessed by Pearson´s correlation.

The reproductive performance of the sows used in the study was analyzed for normality by the Kolmogorov-Smirnov test, which showed that all parameters had non-normal distribution, so, the non-parametric U-Mann Whitney test was used. The results are represented as the mean ± SD (standard deviation). Values were considered significantly different when *p* ≤ 0.05.

Rectal temperature data were analyzed using the Statistic Analysis Software (SAS, University Edition 2016). Sphericity of the data was checked for the temperature of the sows (pre-vaccination, 6 h, and 12 h post-vaccination). All the variables confirmed the sphericity of the data, and they were analyzed using Proc Mixed procedures. The model included the treatments (vaccinated vs. non-vaccinated sows), the three points of evaluation (pre-vaccination, 6 h post-vaccination, and 12 h post-vaccination), and the interaction between these as the main effect, with different samples and sows as the random effect. Data temperatures are represented as the mean ± SD (standard deviation).

The weights of piglets were analyzed for normality by a Kolmogorov-Smirnov test, which showed that all parameters had a non-normal distribution except weight gain from birth to the final weight, piglet weight at the end of the study and ADWG from birth to the final weight. Then, the U-Mann-Whitney test was used for non-parametric parameters and the T-test for parametric parameters. Values were considered significantly different when *p* ≤ 0.05. A statistical trend was considered when *p* > 0.05 and *p* < 0.07. Weight data in the text are represented as the mean ± SD (standard deviation). The feed consumed per sow was analyzed for normality by a Shapiro–Wilk test, which showed that the data had a normal distribution, so a t-student test was applied. Values were considered significantly different when *p* ≤ 0.05. Feed consumed data in the text are represented as the mean ± SD (standard deviation).

## 3 Results

Sows used in experiment 1 (Table 1; Farm A) showed similar characteristics between both experimental groups. Gross examinations did not reveal significant differences between vaccinated and non-vaccinated females based on the weights of the ovaries, uterus, and oviduct.

**Table 1:** Characteristics (number of parities and reproductive tract) of the sows from the two experimental groups (non-vaccinated vs. vaccinated sows). Data are expressed as mean ± SD. The values are the mean of right and left structures (ovary, oviduct, uterine horn, and uterine arteries) and the mean total number of follicles per sow. No significant differences were observed (*p* > 0.05).

	N	Parity	Ovaries weight (g)	Total number of follicles per sow	Oviduct weight (g)	Uterine horns weight (g)	Uterine arteries (cm)
**Non-vaccinated**	9	6.88 ± 0.78	7.83 ± 1.60	67.22 ± 16.02	3.31 ± 0.44	304.20 ± 47.97	0.57 ± 0.07
**Vaccinated**	10	6.60 ± 1.07	7.01 ± 1.63	74.60 ± 18.23	3.26 ± 1.28	309.00 ± 51.90	0.53 ± 0.06
**p-value**		0.235	0.833	0.364	0.116	0.937	0.685

**Fig. 2:**
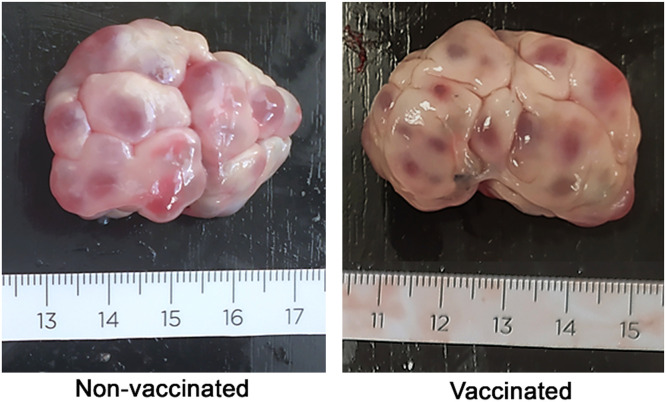
Ovaries of non-vaccinated and vaccinated sows the day after weaning at the beginning of the follicular phase, after lactation anestrus. Different follicle sizes are observed in both groups. Scale in cm.

The frequencies of each follicle size were significantly different between vaccinated vs non-vaccinated sows (*p* < 0.001), with vaccinated sows showing a lesser frequency of large follicles and a higher frequency of small follicles than non-vaccinated sows. When the percentage of follicles in each category (small, medium or large) was compared, a significant higher rate of large follicles was observed in non-vaccinated compared to vaccinated animals (Table 2), (Fig. 2).

**Table 2:** Percentage of small, medium and large follicles per ovary from the two experimental groups (non-vaccinated vs. vaccinated sows). Data are expressed as mean ± SEM. No significant differences were observed (*p* > 0.05). Dots-graph showing the percentage of follicular size [small (represented in blue), medium (represented in grey) and large (represented in green)].

	Ovaries	Small follicles (%,)	Medium follicles (%,)	Large follicles (%,)	Dots-graph
**Non-vaccinated**	18	24.95 ± 4.04	39.36 ± 6.35	35.65 ± 4.97	
**Vaccinated**	20	35.41 ± 5.61	44.71 ± 5.78	19.89 ± 3.39	
**p-value**		0.12	0.33	0.035

No correlation was detected between the size of the follicles and ovary weight (*p* > 0.05), but a positive significant correlation was established between the number of large follicles and the size of the uterine artery (*r* = 0.678; *p* = 0.022). In addition, when this last correlation was performed separately in the two experimental groups, a statistical trend was found for non-vaccinated sows (*r* = 0.872; *p* = 0.054) but correlation was not observed for the vaccinated sows (*r* = 0.323; *p* = 0.533).

At farm B, all animals showed normal reproductive performance prior to the experiments without differences between the experimental groups (Table 3). The vaccination (or placebo in the case of the control group) of the sows was performed after ∼11 days of lactation (11.27 ± 1.52 and 11.22 ± 1.39 days for vaccinated and non-vaccinated sows, respectively; *p* = 0.939).

**Table 3:** Reproductive performance in sows used for both experimental groups (non-vaccinated vs. vaccinated sows). Data are shown as mean ± SD. No significant differences were observed (*p* > 0.05).

	N	Parity	Total piglets born	Piglets born alive	Piglets born dead	Mummified piglets
**Vaccinated**	33	3.85 ± 2.98	14.15 ± 3.40	13.01 ± 3.34	0.97 ± 1.52	0.06 ± 0.37
**Non-vaccinated**	31	3.88 ± 2.80	13.98 ± 3.68	12.63 ± 2.81	1.30 ± 1.90	0.14 ± 0.60
**p-value**		0.927	0.877	0.481	0.265	0.345

In experiment 2, the sows’ rectal temperature during the trial (pre- and post-vaccination) did not display any differences between vaccinated and non-vaccinated sows (*p* > 0.05). Moreover, no adverse local reactions were observed in any of the investigated groups.

**Fig. 3:**
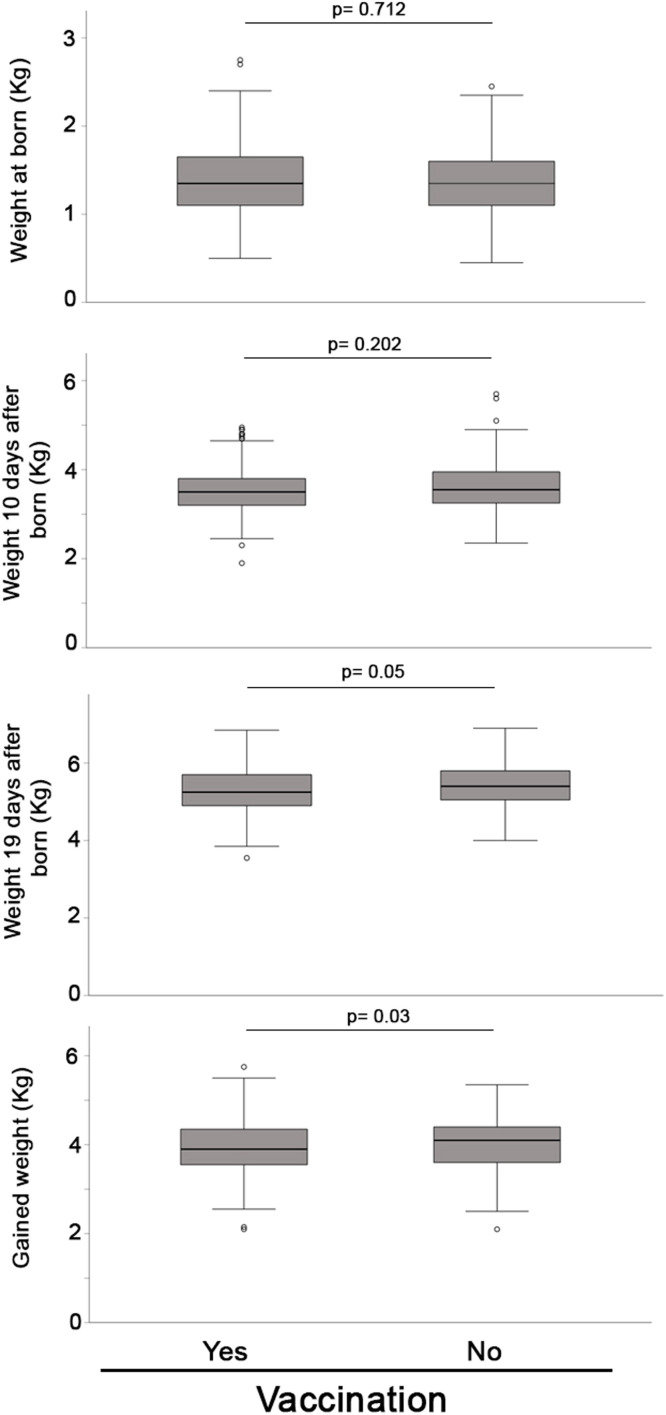
Box-plot comparing the weight (kg) (at born, after 10 days *n*= and around 19 days after born, and total gained weight (*n* = 221 and *n* = 158 in vaccinated and non-vaccinated group respectively) of the piglets born in the two groups of sows (non-vaccinated vs. vaccinated). Significant differences were observed when *p* < 0.05. Small circles (°) and asterisks (*) in the box-plot represents outliers and extreme cases, respectively.

In experiment 3, the number of lactation days until the final weight studied were 19.18 ± 1.34 and 19.25 ± 1.43 days for vaccinated and non-vaccinated sows respectively (*p* = 0.617). The weights of piglets between groups at birth and at 10 days of lactation were similar (1.39 ± 0.39 and 1.37 ± 0.37 kg for piglets from vaccinated and non-vaccinated sows at birth; *p* = 0.712 and 3.54 ± 0.54 and 3.64 ± 0.59 kg for piglets from vaccinated and non-vaccinated sows at 10 days; *p* = 0,202). Statistical differences were observed at final weight near weaning (5.28 ± 0.57 and 5.40 ± 0.56 kg for piglets from vaccinated and non-vaccinated sows, respectively; *p* = 0.05). When the total weight gained per piglet between birth and the final weigh was analyzed, the results indicated a significant improvement for the piglets from non-vaccinated sows (3.89 ± 0.62 and 4.02 ± 0.60; for vaccinated and non-vaccinated sows respectively, *p* = 0.03) (Fig. 3).

ADWG during the studied period showed a statistical trend in favor the non-vaccinated group (0.204 ± 0.032 and 0.210 ± 0.034; for vaccinated and non-vaccinated sows respectively, *p* = 0.06).

**Fig. 4:**
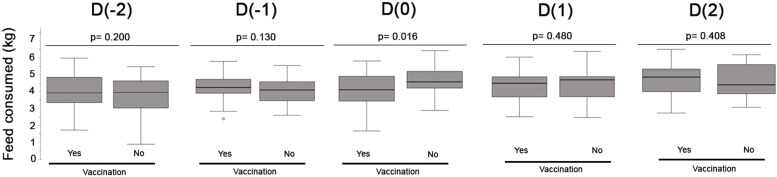
Box-plot comparing the feed consumed per sow (kg) the 2 days previous to vaccination (D(−2) and D(−1)), the day of vaccination (D(0)) and 2 days after vaccination (D(1) and D(2 in the two groups of sows (vaccinated vs. non-vaccinated). Significant differences were observed when *p* < 0.05. Small circles (°) in the box-plot represents outliers and extreme cases, respectively.

When the feed consumed per sow (experiment 4) was analyzed the 2 days previous to vaccination (D(−2) and D(−1)), the day of vaccination (D(0)), and 2 days after vaccination (D(1) and D(2)), a significant difference was observed the D0 (*p* = 0.016) showing a lower feed consumption per sow in the vaccinated group compared to the non-vaccinated group (4.09 ± 1.03 and 4.62 ± 0.82 kg, Fig. 4).

## 4 Discussion

Successful swine production aims to deliver as many live-born and healthy piglets as possible by, among other factors, preventing infectious diseases affecting reproductive output. Vaccination is one of the most successful and cost-effective interventions to prevent swine diseases such as erysipelas and parvovirus. However, vaccination strategies on farms should be established to achieve not only an adequate level of immunity against pathogens but also to decrease the stress of the animals in critical periods of swine production to maximize its production and welfare. Our study has shown the effect of the administration of a combined vaccine of ER and PPV during the lactation period on reproductive and productive efficiency.

Few studies have analyzed the putative impact of vaccination on the reproductive tract and ovary function, except those related to ovarian regulation (Jinshu et al., 2005; Wang et al., 2012) or contraception (Ma et al., 2012; Dalmau et al., 2015) through vaccines. First, we compared the effect of vaccination during day 11 of lactation on the ovary and reproductive tract. The study demonstrates that this ER/PPV vaccine has no impact on the female reproductive tract and the total number of follicles in the ovaries did not vary between non-vaccinated and vaccinated sows. This fact agrees with a previous report comparing PRRSV -vaccinated and non-vaccinated sows concerning gross measurements of ovaries and uterus (Papatsiros et al., 2015). However, our results indicated that the non-vaccinated sows showed a higher frequency of larger follicles than vaccinated sows. In pigs, some studies have shown, that developmental competence of pig oocytes improves as follicular size increases (Marchal et al., 2002). To the best of our knowledge, this is the first study analyzing the impact of vaccination on porcine follicular size. This is of paramount importance because sows showing smaller follicles at weaning had showed longer intervals for estrus and ovulation and smaller litters at farrowing (TP Lopes et al., 2020). Lopes et al. also stated that differences in follicles size in the early stage of lactation persisted throughout the entire period and weaning, reinforcing the notion that vaccinations and stress should be minimized during this stage (TP Lopes et al., 2020).

In our study, a positive correlation was also found between large follicle proportion and uterine artery size in the non-vaccinated group. Although in another species the positive correlation observed between ovarian follicular size and uterine artery vascular indices was demonstrated in cows (Yama et al., 2022).

The populations of follicles observed in the present study agree with the physiology of the ovary during lactation. Small and medium-sized follicles (no larger than 3 mm) are present during the second week of lactation for a later increase in their size because of a higher frequency of luteinizing hormone (LH) pulses but not developed beyond 5 mm in diameter during lactation (Quesnel, 2009). One aspect to consider is the impact of feed reduction intake on the day of vaccination on follicular development. Our results indicated a reduction of follicle size population in the vaccinated sows and a significant decrease in feed intake on the day of vaccination in this group of animals. It has been demonstrated that gilts deprived of glucose reduced LH pulse frequency (Barb et al. 2001) which may be related to the reduction in the follicular growth observed in our study. Restriction feed to sows were reported to inhibit secretion of LH and result in fewer follicles larger than 4 mm at weaning and two days later (Quesnel et al., 1998). So, nutritional deficiency during lactation can alter follicular growth.

Overall, the administered vaccine did not induce a significant increase in the mean rectal temperature compared to non-vaccinated animals. Furthermore, none of the animals of the study had a rectal temperature increase above 1.3 °C (including vaccinated and non-vaccinated animals) after injection except two animals injected with the vaccine which had an increase of 1.9 °C. These findings agree with previous reports using the same bivalent vaccine in pregnant gilts (Noguera et al., 2021) or different PPV vaccine in lactating sows (García-Morante et al., 2019).

The number of pigs weaned per sow is commonly used as a measurement to benchmark the productivity of breeding herds (Koketsu et al., 2017). However, other factors such as piglet quality should be considered. A previous study using a different vaccine showed no differences between the vaccinated group and non-vaccinated regarding ADWG in piglets (García-Morante et al., 2019), and vaccine applied was only against PPV but not ER, and the study was longer than our study (19 vs. 28 days). Although in this study similar to our results a significant difference in piglet weight gain from birth to the final weight at weaning in favor of non-vaccinated group was found. Moreover, the AWDG trend to be higher in the piglets from the non-vaccinated group. This aspect may be associated with the reduced sow feed intake in the period around vaccination, where the feed consumed significantly decreased the day of inoculation in the vaccinated group. Feed intake is a critical point in lactating sows and lower lactation feed intake has been associated with a lower average weaning weight of piglets and reproductive failures (Koketsu et al., 1996) which agrees with our results.

Despite the mild nature vaccine's effects, our study indicates that its administration during lactation can have several adverse consequences on sow physiology and offspring performance, as previously discussed. In this regard, adverse reactions to vaccination may result to the inflammatory response to the antigen or the presence of an adjuvant in the vaccine (Spickler et al., 2003). The alum used as an adjuvant in the vaccine administered in our study can activate cellular transcription factors and stimulate the production of various signaling molecules. For example, it can trigger the Nalp3 inflammasome, leading to a humoral adaptive immune response (IgE production) (Eisenbarth et al., 2008; Kool et al., 2008; Kuroda et al., 2011). Additionally, alum can activate dendritic cells through receptor-independent uptake, induce lipid mediators such as prostaglandins, and stimulate the innate immune system by host DNA released from alum-injured cells (Flach et al., 2011; Kuroda et al., 2011; Marichal et al., 2011). Moreover, high doses of aluminum hydroxide injected without antigens has demonstrated to cause granulomas in pigs (Valtulini et al., 2005). However, alum remains the most widely used vaccine adjuvant due to its safety, cost-effectiveness, and ability to enhance the immune response to various antigens (Di Benedetto et al., 2024). Including an experimental group receiving only the adjuvant could have provided a more comprehensive interpretation of the results. We acknowledge this is a limitation of the present study; however, it is important to note that all commercially available bivalent ER/PPV vaccines come as ready-to-use suspension for injection, and the focus is on evaluating whether the complete product available to veterinarians (adjuvant + antigen) influences reproductive performance.

## 5 Conclusions

This study determined that administering the bivalent ER/PPV vaccine to sows during lactation can have a detrimental effect on follicle development, leading to a reduced frequency of large follicles. Additionally, piglets from vaccinated sows exhibited lower final weight, reduced weight gain, and a tendency toward lower average daily weight gain during lactation. Furthermore, vaccinated sows had lower feed consumption on the day of vaccination compared to the non-vaccinated group.

It is well established that ER and PPV are major contributors to reproductive failure in the swine industry, and vaccination remains the most cost-effective strategy to prevent these diseases worldwide.

The findings of this study highlight the need for further research to evaluate alternative vaccination strategies, such as administration during gestation or as part of a mass vaccination program that reduces vaccine administration during lactation, to mitigate potential negative effects on sow reproductive performance and piglet growth.

## Institutional review board statement

Through the experiments, animals were handled carefully avoiding any unnecessary stress. All experiments were performed following relevant guidelines and regulations. The study was carried out in compliance with the ARRIVE guidelines (https://arriveguidelines.org/). An Internal consultation regarding the need of a full review from an Ethics committee was carried out with two experts in Boehringer Ingelheim: Spanish Regulatory and Pharmacovigilance Head and European Head of Clinical Studies. The ethics approval was deemed unnecessary according to European and national legislation (Directive 2010/63/EU of the European parliament and RD53/2013). In both regulation state that the ethics approval is not necessary in case of: a) non-experimental agriculture practices, b) non-experimental clinical veterinary practices and d) practices undertaken for the purposes of recognized animal husbandry. This study is framed in these assumptions because the procedures carried out are commonly used in the farms.

## Informed consent statement

Ganados Gili, S.A. (Owner of the animals) consented to participate and stated that the study is about the performance impact of current vaccine protocols in their farms. Likewise, the study utilized reproductive tracts obtained from sows that were culled as part of the farm's routine culling process. All their staff involved are trained to use of the best welfare practices in swine production and part of their veterinarians actively monitored and performed the research.

## CRediT authorship contribution statement

**E. Sánchez-Tarifa:** Writing – review & editing, Investigation, Data curation, Conceptualization. **FA. García-Vázquez:** Writing – review & editing, Writing – original draft, Conceptualization. **A. Vela:** Investigation. **L. Sanjoaquín:** Investigation. **MV. Falceto:** Investigation. **A. Fernández-Fontelo:** Formal analysis, Data curation. **O. Gómez-Duran:** Writing – review & editing. **C. Alonso:** Writing – review & editing. **I. Hernández-Caravaca:** Writing – review & editing, Writing – original draft, Conceptualization.

## Declaration of competing interest

The authors declare the following financial interests/personal relationships which may be considered as potential competing interests:

E. Sanchez-Tarifa reports financial support, article publishing charges, and equipment, drugs, or supplies were provided by Boehringer Ingelheim Vetmedica GmbH. E. Sanchez-Tarifa reports a relationship with Boehringer Ingelheim GmbH that includes: employment. O.G. Duran reports a relationship with Boehringer Ingelheim Vetmedica GmbH that includes: employment. C. Alonso reports a relationship with Boehringer Ingelheim Vetmedica GmbH that includes: employment. If there are other authors, they declare that they have no known competing financial interests or personal relationships that could have appeared to influence the work reported in this paper.

## Data Availability

The raw data supporting the conclusions of this article will be made available by the authors on request.

## References

[bib0001] Auvigne V., Leneveu P., Jehannin C., Peltoniemi O., Sallé E. (2010). Seasonal infertility in sows: A five year field study to analyze the relative roles of heat stress and photoperiod. Theriogenology.

[bib0002] Barb C.R., Barrett J., Kraeling R.R., Rampacek GB 2001b. Serum leptin concentrations, luteinizing hormone and growth hormone secretion during feed and metabolic fuel restriction in the prepuberal gilt. Domestic Animal Endocrinology 20 47–63. 10.1016/s0739-7240(00)00088-6 11164333

[bib0003] Bertoldo M.J., Holyoake P.K., Evans G., Grupen C.G. (2011). Seasonal effects on oocyte developmental competence in sows experiencing pregnancy loss. Animal Reproduction Science.

[bib0004] Bilkei G. (1996). Sauenmanagement. Vet spezial. Gustav Fischer Verlag. Jena and Stuttgart.

[bib0005] Buchanan R.E. (1918). Studies in the nomenclature and classification of the bacteria. Journal of Bacteriology.

[bib0006] Dalmau A., Velarde A., Rodríguez P., Pedernera C., Llonch P., Fàbrega E. (2015). Use of an anti-GnRF vaccine to suppress estrus in crossbred Iberian female pigs. Theriogenology.

[bib0007] Di Benedetto R., Massai L., Wright M. (2024). Adjuvanted modified bacterial antigens for single-dose vaccines. International Journal of Molecular Sciences.

[bib0008] Eisenbarth S.C., Colegio O.R., O'Connor W., Sutterwala F.S., Flavell R.A (2008). Crucial role for the Nalp3 inflammasome in the immunostimulatory properties of aluminium adjuvants. Nature.

[bib0009] Flach T.L., Ng G., Hari A. (2011). Alum interaction with dendritic cell membrane lipids is essential for its adjuvanticity. Nature Medicine.

[bib0010] Foxcroft G.R., Hunter M.G. (1985). Basic physiology of follicular maturation in the pig. Journal of Reproduction and Fertility. Supplement.

[bib0011] Garcia-Morante B., Noguera M., Klocke S., Sommer K., Kaiser T., Haist V. (2019). A novel subunit vaccine based on the viral protein 2 of porcine parvovirus: Safety profile in bred pigs at different stages of the reproduction cycle and in offspring. Heliyon.

[bib46] Hafez R.S.E. (2000).

[bib47] Hernández-Caravaca I, Izquierdo-Rico MJ, Matás C (2012). Reproductive performance and backflow study in cervical and post-cervical artificial insemination in sows. Anim Reprod Sci.

[bib0013] Hoffmann C.W., Bilkei G. (2002). Case study: Chronic erysipelas of the sow–a subclinical manifestation of reproductive problems. Reproduction in Domestic Animals.

[bib0014] Holtkamp D.J., Polson D.D., Torremorell M., Morrison B., Classen D.M., Becton L. (2011). American association of swine veterinarians; United States department of agriculture PRRS-coordinated agricultural project. Terminology for classifying the porcine reproductive and respiratory syndrome virus (PRRSV) status of swine herds. Tierarztliche Praxis. Ausgabe G, Grosstiere/Nutztiere.

[bib0012] Jinshu X., Jingjing L., Duan P., Zheng Z., Ding M., Jie W. (2005). A synthetic gonadotropin-releasing hormone (GnRH) vaccine for control of fertility and hormone dependent diseases without any adjuvant. Vaccine.

[bib0015] Kemp B., Soede N.M., Langendijk P. (2005). Effects of boar contact and housing conditions on estrus expression in sows. Theriogenology.

[bib0016] Knox R.V. (2019). Physiology and Endocrinology Symposium: Factors influencing follicle development in gilts and sows and management strategies used to regulate growth for control of estrus and ovulation1. Journal of Animal Science.

[bib0017] Kool M., Pe´trilli V., De Smedt T., Rolaz A., Hammad H., van Nimwegen M. (2008). Cutting edge: Alumadjuvantstimulates inflammatory dendriticcells through activation of the NALP3 inflammasome. Journal of immunology (Baltimore, Md. : 1950).

[bib0018] Koketsu Y., Dial G.D., Pettigrew J.E., King V.L. (1996). Feed intake pattern during lactation and subsequent reproductive performance of sows. Journal of Animal Science.

[bib0019] Koketsu Y., Tani S., Iida R. (2017). Factors for improving reproductive performance of sows and herd productivity in commercial breeding herds. Porcine Health Management.

[bib0020] Kuroda E., Ishii K.J., Uematsu S. (2011). Silica crystals and aluminum salts regulate the production of prostaglandin in macrophages via NALP3 inflammasome-independent mechanisms. Immunity.

[bib0021] Lopes T.P., Padilla L., Bolarin A., Rodriguez-Martinez H., Roca J. (2020). Ovarian follicle growth during lactation determines the reproductive performance of weaned sows. Animals (Basel)..

[bib0022] Lopes T.P., Sanchez-Osorio J., Bolarin A., Martinez E.A., Roca J. (2014). Relevance of ovarian follicular development to the seasonal impairment of fertility in weaned sows. The Veterinary Journal.

[bib0023] Lopes T.P., Padilla L., Bolarin A., Rodriguez-Martinez H., Roca J. (2020). Ovarian follicle growth during lactation determines the reproductive performance of weaned sows. Animals : an open access journal from MDPI.

[bib0024] Lucy M.C., Liu J., Boyd C.K., Bracken C.J. (2001). Ovarian follicular growth in sows. Reproduction Supplement.

[bib0025] Ma X., Li J., Zhang F. (2012). Intranasal codelivery with the mouse zona pellucida 3 and GM-CSF expressing constructs enhances humoral immune responses and contraception in mice. Scandinavian Journal of Immunology.

[bib0026] Marchal R., Vigneron C., Perreau C., Bali-Papp A., Mermillod P. (2002). Effect of follicular size on meiotic and developmental competence of porcine oocytes. Theriogenology.

[bib0027] Marichal T., Ohata K., Bedoret D. (2011). DNA released from dying host cells mediates aluminum adjuvant activity. Nature Medicine.

[bib0028] Morimoto M., Kato A., Nogami K., Akaike Y., Furusawa T., Kojima H. (2022). The swine erysipelas vaccine SER-ME effectively protects pigs against challenge with the *Erysipelothrix rhusiopathiae* M203/I257 SpaA-type variant. Veterinary Sciences.

[bib0029] Noguera M., Vela A., Kraft C., Chevalier M., Goutebroze S., de Paz X. (2021). Effects of three commercial vaccines against porcine parvovirus 1 in pregnant gilts. Vaccine.

[bib0030] Opriessnig T., Forde T., Shimoji Y. (2020). *Erysipelothrix* Spp.: Past, present, and future directions in Vaccine research. Frontiers in Veterinary Science.

[bib0031] Papatsiros V., Psalla D., Papaioannou D., Tassis P., Tzika E., Papaioannou N. (2015). Pathological evaluation of reproductive system of porcine reproductive and respiratory syndrome virus-vaccinated and nonvaccinated anestrus sows and gilts. Viral Immunology.

[bib0032] Peltoniemi O.A., Virolainen J.V. (2006). Seasonality of reproduction in gilts and sows. Society of Reproduction and Fertility supplement.

[bib0033] Quesnel H., Pasquier A., Mounier A.M., Prunier A. (1998). Influence of feed restriction during lactation on gonadotropic hormones and ovarian development in primiparous sows. Journal of Animal Science.

[bib0034] Quesnel H., Boulot S., Le Cozler Y. (2005). Seasonal variation of reproductive performance of the sow. INRA Productions Animales..

[bib0035] Quesnel H. (2009). Nutritional and lactational effects on follicular development in the pig. Society of Reproduction and Fertility supplement.

[bib0036] Sánchez-Matamoros A., Camprodon A., Maldonado J., Pedrazuela R., Miranda J. (2019). Safety and long-lasting immunity of the combined administration of a modified-live virus vaccine against *porcine reproductive and respiratory syndrome virus 1* and an inactivated vaccine against *porcine parvovirus* and *erysipelothrix rhusiopathiae* in breeding pigs. Porcine Health Management.

[bib48] SAS Institute (2016). Statistical Analysis Software (SAS) User’s Guide Version 9.4.

[bib0037] Skerman V.B.D., McGowan V., Sneath P.H.A. (1980). Approved lists of bacterial names. International Journal of Systematic Bacteriology.

[bib0038] Spickler A.R., Roth J.A. (2003). Adjuvants in veterinary vaccines: Modes of action and adverse effects. Journal of Veterinary Internal Medicine.

[bib0039] Stephenson E.H., Berman D.T. (1978). Isolation of *Erysipelothrix rhusiopathiae* from tonsils of apparently normal swine by two methods. American Journal of Veterinary Research.

[bib0040] Streck A.F., Truyen U. (2020). Porcine parvovirus. Current issues in molecular biology.

[bib0041] Truyen U., Streck A.F., Zimmerman J.K., Karriker L., Ramirez A., Schwartz K.J., Stevenson G.W. (2012). Diseases of swine.

[bib0042] Valtulini S., Macchi C., Ballanti P. (2005). Aluminium hydroxide-induced granulomas in pigs. Vaccine.

[bib0043] Van den Born E., van den Elzen P.P.M., van Kilsdonk E., Hoeijmakers M.J.H., Segers R.P.A.M. (2020). An octavalent vaccine provides pregnant gilts protection against a highly virulent porcine parvovirus strain. BMC Veterinary Research.

[bib0044] Wang S.L., Han L., Ahmad S., Cao S.X., Xue L.Q., Xing Z.F. (2012). Effect of a DNA vaccine harboring two copies of inhibin α (1-32) fragments on immune response, hormone concentrations and reproductive performance in rats. Theriogenology.

[bib0045] Yama P., Yadmak C., Sangkate M., Jitjumnong J., U-Krit W., Promsao N. (2022). In vivo follicular and uterine arterial indices as an indicator of successful hormonal stimulation for inactive ovaries in repeat-breeder crossbred dairy cows using a short-term progesterone-based programme. Animals : an open access journal from MDPI.

